# The Use of Gene Modification and Advanced Molecular Structure Analyses towards Improving Alfalfa Forage

**DOI:** 10.3390/ijms18020298

**Published:** 2017-01-29

**Authors:** Yaogeng Lei, Abdelali Hannoufa, Peiqiang Yu

**Affiliations:** 1 Department of Animal and Poultry Science, College of Agriculture and Bioresources, University of Saskatchewan, Saskatoon, SK S7N 5A8, Canada; yal263@mail.usask.ca; 2 Agriculture and Agri-Food Canada, 1391 Sandford Street, London, ON N5V 4T3, Canada; abdelali.hannoufa@agr.gc.ca

**Keywords:** alfalfa, proanthocyanidin and anthocyanins, lignin, genetic modification, transcription factors, molecular structure

## Abstract

Alfalfa is one of the most important legume forage crops in the world. In spite of its agronomic and nutritive advantages, alfalfa has some limitations in the usage of pasture forage and hay supplement. High rapid degradation of protein in alfalfa poses a risk of rumen bloat to ruminants which could cause huge economic losses for farmers. Coupled with the relatively high lignin content, which impedes the degradation of carbohydrate in rumen, alfalfa has unbalanced and asynchronous degradation ratio of nitrogen to carbohydrate (N/CHO) in rumen. Genetic engineering approaches have been used to manipulate the expression of genes involved in important metabolic pathways for the purpose of improving the nutritive value, forage yield, and the ability to resist abiotic stress. Such gene modification could bring molecular structural changes in alfalfa that are detectable by advanced structural analytical techniques. These structural analyses have been employed in assessing alfalfa forage characteristics, allowing for rapid, convenient and cost-effective analysis of alfalfa forage quality. In this article, we review two major obstacles facing alfalfa utilization, namely poor protein utilization and relatively high lignin content, and highlight genetic studies that were performed to overcome these drawbacks, as well as to introduce other improvements to alfalfa quality. We also review the use of advanced molecular structural analysis in the assessment of alfalfa forage for its potential usage in quality selection in alfalfa breeding.

## 1. Introduction

Alfalfa (*Medicago sativa*) is also known as “Queen of Forage” due to its high biomass yield, good forage quality, and palatability for ruminants [1]. Owing to its high adaptability, alfalfa can grow in a wide variety of landscapes and growing conditions. The relatively deep root system enables alfalfa to absorb water from deep sub-soil layer, allowing this plant to flourish in drought areas with only 200 mm annual precipitation [1]. In addition, the symbiotic relationship with *Rhizobia*, the common feature of legume plants, provides nitrogen nutrients for alfalfa, enabling it to grow in nutrient poor soils. Such nitrogen-fixing relationship is also a benefit to soil by improving its structure [2]. Good adaptability, high biomass yield, and high nutritive value make alfalfa one of the most widely cultivated forage crops around the world. According to Tesfaye et al. [3], the growing area of alfalfa is approximate 32 million hectares worldwide. The cultivated area of alfalfa in Canada was 4.5 million hectares in 2011 [4], double the value published in 2009 [5]. Although the growing area has declined to some extent in recent years [6], alfalfa is still an important forage crop in the livestock industry. However, alfalfa is not without problems, and there are two drawbacks that limit its utilization, which will be further discussed below.

### 1.1. Drawbacks that Limit Alfalfa’s Utilization

Despite its high quality, alfalfa protein is poorly utilized [7]. This drawback is mainly due to its high content of soluble crude protein, which can be rapidly degraded in the rumen resulting in waste of high quality protein. In addition, the rapid degradation of alfalfa protein can increase the viscosity of ruminal fluid, posing a risk of pasture bloat to animals [8]. In contrast to alfalfa, some legume forages, such as sainfoin (*Onobrychis viciifolia*), birdsfoot trefoil (*Lotus corniculatus*), and big trefoil (*Lotus pedunculatus*), also have high content of protein but are bloat-free. This anti-bloat character is highly associated with their possession of proanthocyanidins (PAs), which are also known as condensed tannin [7,9,10]. PAs can bind with protein in rumen thereby decreasing its degradation, and reducing the risk of rumen bloat [11]. A moderate amount of PAs in forages, about 2%–4% of dry matter (DM), can slow down protein degradation rate in the rumen, increase rumen escape protein, and improve animal productivity [12]. However, there are only trace amounts of PAs in alfalfa, and they are restricted to the seed coat [13,14].

Another drawback of alfalfa is its relatively high lignin content [15]. As the second most abundant component in secondary cell walls [16], lignin is of great importance for plant growth, development, and pathogen resistance [17]. Many of the genetic modifications introduced to plants to reduce lignin content have resulted in dwarfed phenotypes, which have led to compromised biomass yield, limiting the benefits of reduced lignin [18]. However, lignin is resistant to digestion and degradation and is hardy useful to animals, even to ruminants. There are only a few microorganisms that are able to degrade lignin, including some fungi and bacteria, by using extracellular enzymes like lignin peroxidase, manganese peroxidase, and versatile peroxidase [19,20]. However, this degradative process is an oxidative reaction requiring an aerobic environment. In the anaerobic environment of rumen, the degradability of lignin is limited and negligible compared to that of cellulose and hemicellulose [21]. Moreover, lignin also hinders the degradation of other compounds by its inhibitory effects on cellulolytic enzymes and crosslinks with polysaccharides [22,23,24]. In the production of biofuel, a thermochemical pretreatment is required to eliminate or delocalize lignin prior to the enzymatic hydrolysis of polysaccharides into fermentable sugars [20].

### 1.2. Biosynthesis of Proanthocyanidins and Anthocyanins

**Figure 1:**
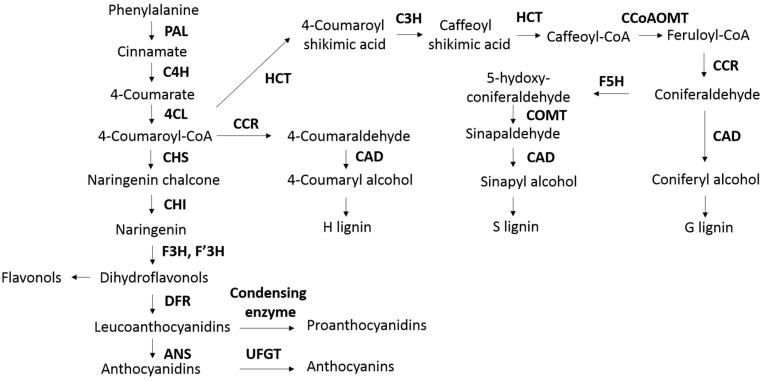
Biosynthesis of proanthocyanidins/anthocyanins and lignin monolognols. This figure was made based on information from Zabala et al. [25], Nesi et al. [29], Vanholme et al. [30] and Jonker [13]. PAL, l-phenylalanine ammonia-lyase; C4H, cinnamate 4-hydroxylase; 4CL, 4-coumarate coenzyme A ligase; CCR, cinnamoyl coenzyme A reductase; CAD, cinnamyl alcohol dehydrogenase; HCT, hydroxycinnamoyl-CoA: shikimate/quinate hydroxycinnamoyltransferase; C3H, coumarate 3-hydroxylase; CCoAOMT, caffeoyl CoA 3-*O*-methyltransferase; F5H, ferulate 5-hydroxylase; COMT, caffeic acid 3-*O*-methyltransferase; CHS, chalcone synthase; CHI, chalcone isomerase; F3H, flavanone 3-hydroxylase; DFR, dihydroflavanol 4-reductase; ANS, anthocyanidin synthase; UFGT, UDP-flavonoid glucosyltransferase. H lignin, hydroxyphenyl; S lignin, syringyl; G lignin, guaiacyl.

Proanthocyanidins (PAs) are a group of secondary metabolites derived from phenylpropanoids, and are the final products of the flavonoid pathway. The biosynthesis pathway of PAs and anthocyanins is shown in Figure 1. Briefly, phenylalanine is first deaminated and then converted into 4-coumaroyl-CoA, which can be either used for lignin synthesis or for flavonoid synthesis. In the PAs synthesis, 4-coumaroyl-CoA is transformed into naringenin after two consecutive reactions. Naringenin can then be hydroxylated to form 5′ OH eriodictyol and eriodictyol, which along with naringenin, are hydroxylated and reduced into three forms of anthocyanidins, namely delphinidin, pelargonidin and cyanidin [25]. After that, these anthocyanidins can be converted to their corresponding anthocyanins through glycosylation [25]. Naringenin in this pathway can also be used to synthesize flavones and isoflavones following a series of reactions.

In addition to structural proteins (enzymes) that catalyze PAs biosynthesis, regulatory factors (transcription factors) also regulate this pathway. Three transcription factors, R/B-like bHLH (basic helix-loop-helix) R2R3-MYB (MYB proto-oncogene), and WD40-repeat proteins, have been reported to regulate the biosynthesis of PAs and anthocyanins [26]. To exert their regulatory function, MYB, bHLH, and WD40 proteins combine with each other forming a tri-protein complex (MBW). MBW controls the expression of late biosynthesis genes in the phenylproponoid pathway, thereby regulating the synthesis of PAs and anthocyanidins [27]. MicroRNA156 (miR156) was shown to enhance anthocyanin biosynthesis in *Arabidopsis thaliana* by silencing *SPL9* (Squamosa promoter binding protein-like9), which acts as a repressor of anthocyanidin biosynthesis by destabilizing the MBW complex [28].

### 1.3. Biosynthesis of Lignin Monomers

Like anthocyanins and PAs, lignin is also derived from the phenylproponoid pathway. Lignin consists mainly of three monolignols: hydroxyphenyl (H lignin), guaiacyl (G lignin), and syringyl (S lignin). The major differences between these monolignols are the degree of the hydroxylation and methylation. The proportion of these monolignols in lignification varies substantially among species and tissues. In angiosperm species, including *Medicago sativa* (alfalfa), lignin consists of mainly G and S monomers with only traces of H monomer, while in gymnosperms lignin consists of a large proportion of G with low S lignin monomers [31]. The proportion of monolignols composing lignin affects the digestive property of the resulting lignin. In paper production, wood containing high G monomer proportion is more resistant to chemical and physical treatment in lignin removal, and an increase in S/G ratio by genetic modification can improve pulping efficiency [32]. This was attributed to the fact that abundance of G monomers leads to a more cross-linked than compared to that rich in S monomers [32].

The biosynthesis of monolignols involves a series of hydroxylation and methylation reactions (Figure 1), and shares the initial three reactions up to 4-coumaroyl-CoA with anthocyanins and PAs pathway [29]. Downstream of 4-coumaroyl-CoA, the pathway splits into two branches leading to either H lignin, or S and G lignin monomers. For G lignin synthesis, only 3-site on the benzol ring is hydroxylated and methylated while S lignin requires hydroxylation and methylated of both 3- and 5-sites. After their biosynthesis in the cytoplasm, monolignols are transported to the cell wall, where they are polymerized by radical-coupling [18,32].

## 2. Genetic Engineering for Improvement of Alfalfa

Conventional breeding for quality improvement in alfalfa is based on phenotypic selection, which has been proved to be time-consuming [33]. Alfalfa breeding is hampered by its allogamous reproductive behavior and inbreeding [34]. More pronounced advances in alfalfa improvement have more recently made use of recombinant DNA technology to modulate expression of genes involved in forage nutritive value, biomass production, and stress tolerance [35,36]. This technology allows for the transfer of DNA sequences of interest into the plant genome to either enhance or silence the expression of target genes that determine important traits in alfalfa and other forage crops [37].

### 2.1. Improvement of Forage Quality

#### 2.1.1. Enhanced Proanthocyanidins Accumulation

As PAs have been shown to prevent pasture bloat and reduce intestinal parasites in ruminant animals [12,38,39], efforts have been made to enhance PAs accumulation in alfalfa. One of the targets for enhancing PAs has been the MBW complex that regulates biosynthesis of PAs and anthocyanins [26]. To increase the accumulation of PAs and anthocyanins in alfalfa, Ray et al. [40] expressed three flavonoid regulatory maize genes *C1*, *Lc* and *B-Peru*, which encode MYB-like protein, bHLH-like protein and bHLH-like structure proteins, respectively. The results showed that only *Lc-*expression alfalfa accumulated higher amounts of PAs and anthocyanins under high intensity light or low temperature [40,41], which implies this accumulation of PAs and anthocyanins is both environment- and gene-dependent. Interestingly, the red color induced by light/temperature stresses faded quickly after removing stresses in greenhouse condition, while in field condition it lasted months, thereby implying that natural light outdoors is sufficient in triggering stress-induced PAs accumulation [40]. In vitro ruminal fermentation of *Lc*-transgenic alfalfa showed a reduced initial degradation rate of DM and nitrogen compared with its non-transgenic parental alfalfa, but with no detectable negative effects on overall degradation [42].

The progenies of *Lc* transgenic alfalfa were obtained by crossing to several commercial alfalfa cultivars, namely Rangelander, Rambler, and Beaver, that were locally grown in western Canada [13]. Compared with their non-transgenic parents, the crossed progenies accumulated more anthocyanins with an average of 197.4 µg/mg DM, contained 3% less crude protein (CP) with 3% more carbohydrates (CHO), reduced the effective degradability and fermentation rate in the rumen, increased the nutrient availability to animals, and reduced foaming properties [7,13,43]. Based on these results, attempts are currently being made to co-express *C1* and *Lc* genes in alfalfa to further boost the PAs and anthocyanins content in alfalfa [41]. Ectopic expression of *R2R3-MYB* gene also have been studied in alfalfa. Hancock et al. [44] overexpressed the *Trifolium arvense* MYB14, a R2R3-MYB transcription factor, in alfalfa and white clover, and found an increase in concentration of PAs up to 1.8% of DM. Verdier et al. [38] reported that expression of the *Medicago truncatula PAR* gene in alfalfa resulted in enhanced PAs accumulation in alfalfa leaves.

#### 2.1.2. Altered Lignin Content and Composition

Most of the research to decrease lignin content in alfalfa has focused on down-regulating key genes in the lignin biosynthesis pathway. Reddy et al. [31] down-regulated cinnamate 4-hydroxylase (*C4H*) through antisense expression in alfalfa, which result in more than a 7-fold reduction in lignin content as determined by the thioacidolysis method, but with no significant changes in lignin composition. In the same study, downregulation of coumarate 3-hydroxylase (*C3H*) resulted in a 3-fold reduction of lignin content with enriched H lignin, while downregulating ferulate 5-hydroxylase (*F5H*) slightly increased G lignin and reduced S lignin. Shadle et al. [45] down-regulated hydroxycinnamoyl CoA: shikimate hydroxycinnamoyl transferase (*HCT*), and found it caused severely reduced G and S lignin monomers and enhanced H lignin. This might be because HCT is involved in 3-hydroxylation by adding and removing a shikimate group, and 3-hydroxylation is a crucial step in S and G lignin synthesis (Figure 1). The *HCT*-silenced transgenic alfalfa plants exhibited some pleotropic effects, including reduced biomass yield, delayed flowering, and impaired vascular structure [45]. Concomitant downregulation of *HCT* and *C3H* produced similar results in terms of lignin content to silencing *HCT* alone, except for a pronounced increase (86.1%) in cellulose content [17]. The author attributed the increase in cellulose content to the genetic variability, growth stage of alfalfa, and the measurement of cellulose. Guo et al. [46] down-regulated caffeic acid 3-*O*-methyltransferase (*COMT*) and caffeoyl CoA 3-*O*-methyltransferase (*CCOMT*) in alfalfa resulting in up to 30% and 18% of reductions in lignin content. However, the reasons for decreased lignin content were different in these two cases, as strong downregulation of COMT caused a substantial loss in S lignin, while strong downregulation of CCOMT resulted in a striking reduction in G lignin. Significant increases in DM digestibility and in vitro gas production have been found in lignin-reduced alfalfa resulting from *COMT* and *CCOMT* down-regulation [47]. In some instances, silencing of lignin biosynthesis genes resulted in changes in lignin composition (lignin monomer ratio) with no effect on total lignin content [32]. Baucher et al. [48] reported that silencing of cinnamoyl alcohol dehydrogenase (*CAD*) decreased S lignin content in alfalfa, but had no effect on total lignin content. This lack of changes in lignin content could be attributed to the increase in other units in lignin polymerization, as larger content of aldehydes have been found in many *CAD*-silenced plants [48]. The S/G ratio could be reduced without having an effect on total lignin content by silencing *COMT* to lower than 20% of wild type [46]. However, the relationship between S/G ratio and forage degradability is controversial, as some transgenic lines with reduced S/G ratio had higher DM disappearance while others did not [48]. Reducted S/G ratios resulting from *F5H*-downregulation also did not improve in vitro DM degradation; in contrast, this *F5H*-downregulated alfalfa had slightly lower DM degradability, which might be due to its slightly higher lignin content [31]. Silencing of *COMT* and *CCOMT* decreased lignin content by reducing the proportion of S monomers and G monomers, respectively, and both of them increased DM digestibility. However, greater improvement of feed quality was achieving by silencing *CCOMT* than by silencing *COMT,* but with similar lignin content [49]. This implies that lignin composition might also affect forage digestibility.

Although reduction in lignin content and/or alteration of lignin composition in alfalfa by genetic modification increased forage digestibility [17,47,49], such genetic modifications were often associated with undesirable traits, such as dwarf phenotype, resulting in huge biomass loss and limiting the benefit of increased digestibility [18]. These pleotropic effects could be the result of loss of lignin itself or other related effects, such as accumulation of harmful intermediates, activation of cell wall integrity pathways, or increased susceptibility to biotic and abiotic stresses [18]. A promising finding was recently reported by McCaslin et al. [50], in which a lignin-reduced alfalfa cultivar developed by Feed Genetics International (FGI), named HarvXtra™, had a normal (not dwarfed) phenotype, and showed an increase in neutral detergent fiber (NDF) digestibility (12%–15%), along with delay in flowering time allowing for a more than seven-day-delay in harvest without sacrificing NDF digestibility.

#### 2.1.3. Improving Other Nutrient Profiles

Modification of expression of target genes has also proven effective in enriching alfalfa with some high value nutrients. For example, Gebril et al. [51] constitutively expressed the maize sucrose phosphate synthase (*SPS*) gene in alfalfa resulting in increased growth rate and enriched crude protein content in leaves. SPS catalyzes the key reaction in the synthesis of plant sucrose, which is a main stable energy product from photosynthesis. Overexpression of SPS in alfalfa increased chlorophyll content and photosynthetic rates and increased the sucrose level in leaves, as well as root mass and nodule numbers [51]. In another study, simultaneously expressing two bacterial genes, aspartate kinase (*AK*) and adenylylsulfate reductase (*APR*), affected amino acid accumulation in alfalfa leading to an increase in levels of sulfur amino acids [52]. AK phosphorylates aspartate, producing the substrate for the synthesis of lysine, threonine and methionine from aspartate. APR reduces sulfate into sulfite for sulfate assimilation, providing reduced sulfur for the synthesis of sulfur-containing compounds. Co-expression of these two genes increased the synthesis of sulfur amino acids, and increased other essential amino acids (EAA), such as lysine and aspartate. These results are very promising for the livestock industry as sulfur amino acids are of great importance for animal performance and production, especially for fiber growth [53]. Accumulation of antioxidant nutrients, such as carotenoids, could increase the tolerance of plants to abiotic stress, which allows dual-improvement in both nutrient value and stress resistance [54]. Wang et al. [2] expressed *ibOr* (sweetpotato orange gene, involves in carotenoid synthesis) in alfalfa resulting in an increase in carotenoid levels, and an improved tolerance to abiotic stresses, including oxidation, salinity, and drought tolerance.

### 2.2. Improvement in Biomass Yield

Efforts have been made to improve alfalfa forage yield through conventional breeding, but with very limited success [55,56]. More recently, molecular-based approaches to yield improvement have been attempted. For example, miR156 is a plant microRNA that has been characterized in many plant species [57]. Overexpression of miR156 in alfalfa caused transcript cleavage-based silencing of seven Squamosa promoter binding protein-like (*SPL*) genes, and eventually enhanced biomass production and shoot branching, delayed flowering time, and improved forage quality characterized by reduced lignin and enhanced cellulose contents [58,59,60]. The delay in flowering onset can prolong the vegetative growth time, thereby increasing the biomass yield per harvest without compromising the nutritive value, which is a similar effect to that of HarvXtra™ alfalfa [50]. This is because, during floral transition, the plants switch from vegetative growth to reproductive growth and repartition their energy supply and photoassimilate to support reproductive development [61,62]. As alfalfa was approaching maturity during this transition, the CP content and forage degradability decrease along with an increase in unavailable protein fractions [63], which could be attributed to the decrease in leaf to stem ratio [64] and lignin accumulation in stems [62]. MiR156 is also involved in regulating biosynthesis of anthocyanidins. Over-expression of miR156 in *Arabidopsis* resulted in an increased level of anthocyanins by repressing the expression of *SPL9* gene [28]. Improving the ability of nutrient absorption can also increase biomass yield under certain conditions. For example, Ma et al. [65] expressed phytase and acid phosphatase genes in alfalfa to improve phosphorous utilization. Compared to the control, transgenic alfalfa had a 2-fold increase in biomass yield in natural soil without phosphorous supplement, and there was a 3-fold increase in biomass when phytate was the only source of phosphorous.

### 2.3. Improvement in Abiotic Stress Resistance

Gene modification and transformation were also used to enhance the tolerance of forage crops to abiotic stress conditions, such as cold, salt drought, and soil pollution. Nie et al. [66] found that transgenic alfalfa with enhanced expression of *Ammopiptanthus mongolicus* dehydrins (*AmDHN*) gene exhibited more resistance to cold stress (4 °C). Some drought-related genes can enhance the drought and salt tolerance of alfalfa. Expression of wheat NHX antiporter gene, *TaNHX2*, in alfalfa resulted in an increase in salt tolerance [67,68]. Tang et al. [69] increased both salt and drought tolerance of alfalfa by overexpressing *Glycine soja WRKY20*, which was to enhance drought tolerance in *Arabidopsis* [70]. In addition to increasing salt and drought tolerance, overexpression of alfalfa GDP-mannose 3′,5′-epimerase (*GME*) gene in *Arabidopsis* resulted in additional acid tolerance, which was attributable to the increment of ascorbate accumulation [71]. This enhanced abiotic tolerance has the potential to be used in phytoremediation. Wang et al. [72] expressed 2,3-dihydroxybiphenyl-1,2-dioxygenase (*BphC.B)* gene, which encodes a key enzyme in catabolism of aromatic compounds, in alfalfa. This heterologous expression resulted not only in an increase in tolerance to polychlorinated biphenyls (PCBs) and 2,4-dichlorophenol (2,4-DCP), but also in a dissipation of these two contaminants in soil. Expression of *P-ATZA*, a bacterial atrazine chlorohydrolase, in tall fescue and alfalfa increased their resistance to atrazine, which was converted into hydroxyatrazine in transgenic plants [73]. More recently, overexpression of miR156 in alfalfa enhanced drought resistance, as evidenced by a higher survival rate, increased root growth, reduced water loss, and increased accumulation of antioxidants, ABA and compatible solutes [74]. Expression of *SPL13* and other drought responsive genes, including *PP2C*, *NCED3*, *P5CS* and senescence-associated gene, was affected in miR156 overexpression alfalfa in response to drought. In this study it was also reported that drought tolerant phenotypes were generated by *SPL13*-silencing, which indicates that miR156 regulates drought tolerance by silencing *SPL13* [74]. The enhanced tolerance to abiotic stresses could make alfalfa more adaptable, enabling alfalfa to grow in more diverse conditions.

In addition, alfalfa has also been used as a platform in molecular farming to produce desirable proteins. For example, Stefanova et al. [75] transformed human lactoferrin gene into alfalfa and successfully detected its expression in leaf tissue. Ferradini et al. [76] used alfalfa as a model plant to enhance the accumulation of heat shock protein 70 (HSP70) for therapeutic purposes. The use of transgenic alfalfa plants to produce vaccines has also been demonstrated [77,78,79].

## 3. Advanced Structural Analyses for Quality Assessment

Gene modification for improving alfalfa quality and stress tolerance could induce molecular structural changes on a molecular basis. Transformation of *Lc*-gene to alfalfa, aiming to enhance the accumulation of PAs and anthocyanins, decreased model-fitted α-helix and β-sheet and increased other protein structures [80]. This result indicates that protein structure varies between transgenic and non-transgenic alfalfa, which could have an influence on their protein nutrient availability. This is because molecular structures of feedstuff are closely related to the nutritive value of forage for animals. Yu et al. [81] reported that high possessions of β-sheet could reduce the degradability of protein, and an increase in β-sheet/α-helix ratio by heat process was associated with an increase in PC protein fraction, which is the undegradable protein in Cornell Net Carbohydrate and Protein System (CNCPS).

The molecular structural changes induced by gene modification are detectable by advanced structural analytical techniques. Such structural analysis can provide various information on molecular chemistry of tested samples and these structural data can be linked to forage quality and nutrient availability to animals [82]. Such linkage between molecular structure and forage quality makes structural analysis a promising tool in forage breeding for quality selection. Moreover, optical structural analysis is more rapid and cost-effective than conventional wet chemical analysis, and it requires less sample amount and less preparations/pretreatment. These advantages could reduce the selection time in breeding cycle. To date, there are three common methods used to analyze molecular structures of feedstuffs: near infrared reflectance spectroscopy (NIRS), Fourier transformed infrared spectroscopy (FTIR), and synchrotron radiation-based IR microspectroscopy (SR-IMS).

### 3.1. Near Infrared Reflectance Spectroscopy

Near infrared reflectance spectroscopy (NIRS) was initially used to measure moisture in grains, fruits and vegetables in 1960s [83,84,85]. Since then, this rapid and cost-effective analytical tool has been well-established as a means for analyzing food and feed samples in terms of components content [86,87,88,89]. NIRS measures the reflectance and absorption of light in the 700–2500 nm region depending on rotational and vibrational energies associated with H-bonding, like O–H, N–H and C–H [90,91,92]. This dependent relationship on H-bonding implies no inorganic minerals can be directly measured by NIRS as they don’t absorb energy in NIR region [91]. However, through their associations with organic matters, some minerals can still be accurately predicted by NIRS. Halgerson et al. [91] reported that accurate prediction of K, Ca and P could be achieved by NIRS, while Mg, S and other micro minerals like Fe, Mn and Si were poorly predicted [91].

### 3.2. Fourier Transformed Infrared Reflectance Spectroscopy

Unlike dispersive spectroscopy using a monochromatic beam, Fourier transformed infrared reflectance spectroscopy (FTIR) uses a polychromatic beam as a light source. This polychromatic beam is initially interfered by an interferometer, which contains a configuration of mirrors, to generate different combinations of light. After measuring the absorption of various combinations of light, the raw absorption data are analyzed in silico through mathematical procedures, Fourier transform [93], which is the main characteristic of FTIR. The light used by FTIR can have a wide range of wavelengths, including near-IR, mid-IR, and far-IR. This technique has been used in several studies [94,95], including nutritional-related structure study of alfalfa. Marković et al. [96] evaluated the lignin profiles in alfalfa leaves and stems at different maturity stages using attenuated total reflectance (ATR)-FTIR spectroscopy, and found differences between leaves and stems, as leaf lignin mainly consisted of G lignin while stem lignin contained both G and S lignin. Another study conducted by Yari et al. [64] investigated the protein structure of alfalfa at three different maturity stages (early bud, late bud, and early flower) by using FTIR spectroscopy, as well as the relationship between structural profiles and nutrient availability to ruminants. FTIR spectroscopy detected a decrease in the α-helix to β-sheet ratios of protein secondary structures as alfalfa approached maturity, which was negatively related to nutrient supply to ruminants. The studies of Badhan et al. [97] and Jonker et al. [43] showed the capability of FTIR spectroscopy in exploring the structural changes in alfalfa induced by gene modification.

### 3.3. Synchrotron Radiation-Based Fourier Transform IR Microspectroscopy

When FTIR spectroscopy was combined with microscopy and synchrotron radiation, a new analytical tool was created, which is known as synchrotron radiation-based Fourier transform IR microspectroscopy (SR-IMS) [82]. Unlike traditional wet techniques, SR-IMS is capable of analyzing tissues at the cellular and molecular levels without destruction of inherent structures due to the high brightness of synchrotron light, which is much brighter than those used in NIRS and FTIR. Taking advantage of its high brightness light, SR-IMS is capable of detecting the concentrations and specific chemical structures of macromolecular molecules, like proteins, lipids, carbohydrates, and nucleic acids.

To date, this advanced technique has been used to explore the intrinsic molecular structures of various feedstuffs. By using this technique, Yu et al. [82] successfully obtained the nutritional information of barley in terms of spectral and chemical characteristics and biological components distribution. Other feedstuffs like oat [98], corn [99], wheat [100], and dried distillers grains with solubles (DDGS) [101] were also analyzed using SR-IMS at the National Synchrotron Light Source in Brookhaven National Laboratory (NSLS-BNL), Advanced Light Source in Berkeley National Laboratory (ALS-BNL) and/or Canada. Light Source (CLS) synchrotron Centers. This advanced technique is also capable of exploring structural changes induced by feed processing. According to the results of Doiron et al. [102], heating time had an influence on molecular structures of flaxseeds, and such influence affected its rumen degradation. Zhang and Yu [103] applied this bioanalytical technique to identify molecular changes in response to heat-processing in different types of oil seeds, and found that moisture heating had a greater impact than dry heating in terms of the penetration of heat into the intrinsic tissue.

Molecular structural changes in alfalfa tissue induced by gene transformation have also been explored by using molecular structural analytical techniques [15,80,104,105]. Li et al. [15] explored the effects of silencing *TT8* and *HB12* by RNAi on the chemical profile and carbohydrate structure in alfalfa. Results showed that silencing of *TT8* and *HB12* increased NDF content and NDF digestibility, but had negative influences on the digestibility of rapidly degradable CHO and total CHO in rumen. It was reported that the carbohydrate structural changes induced by gene silencing were closely related to the nutritive value of transgenic alfalfa, such as the bioenergy value and fractions of carbohydrate determined using CNCPS system [105].

## 4. Summary, Implications, and Further Research

Genetic engineering is a promising tool for targeted improvement of forage quality. Recently, numerous studies have been conducted to improve alfalfa’s nutritive value, biomass yield, and stress resistance, as well as to use alfalfa as a platform in molecular farming to produce vaccines and other desirable proteins. In terms of nutritive value of alfalfa, more research is needed to further boost the accumulation of PAs and anthocyanins as current levels are not adequate for bloat prevention. Moreover, gene modification could induce molecular structural changes in alfalfa, and such structural changes are closely correlated with the nutritive value and nutrient availability for animals. Advanced structural analytical techniques could be used as a rapid tool for the assessment of forage quality of newly modified alfalfa lines. Theses advanced analytical tools for measuring structural changes were well-developed and proven to be powerful in analyzing the nutritive value of genetically modified alfalfa. By measuring the reflectance and absorption of infrared light, it is possible to not only explore the concentration and distribution of chemical components, but also image their secondary structural characteristics and molecular structure ratios. More studies are needed to further establish correlations between molecular structure and nutrient degradation and availability for animals.
